# Seven years of pig slurry fertilization: impacts on soil chemical properties and the element content of winter barley plants

**DOI:** 10.1007/s11356-022-21030-2

**Published:** 2022-05-31

**Authors:** Awais Shakoor, Àngela D. Bosch-Serra, José Ramón Olarieta Alberdi, Carmen Herrero

**Affiliations:** 1 Department of Environment and Soil Sciences, University of Lleida, Avda. Alcalde Rovira Roure 191, 25198 Lleida, Spain; 2 Ministry of Climate Action, Food and Rural Agenda, Catalan Government, Avda. Alcalde Rovira Roure 191, 25198 Lleida, Spain

**Keywords:** Barley, Copper, Crop biomass, Phosphorus, Zinc

## Abstract

Intensive pig farming produces large amounts of slurry, which is applied to agricultural soils as fertilizer. A 7-year field study was performed to check the effect of pig slurry on soil properties and on the accumulation of some essential nutrients and heavy metals in a calcareous silty-loam soil (0–0.3 m) and in barley (*Hordeum vulgare* L.) plants in two cropping seasons with contrasting amounts of rainfall. Five fertilization treatments, control (no N applied), mineral fertilizer (90 kg N ha^−1^), and different N doses of pig slurry (146, 281, 534 kg N ha^−1^), were applied at sowing of a barley crop. Organic carbon, available P and K, and total P in soil increased with slurry dose. No differences were found in Co, Cr, Fe, Mn, Ni, and Pb soil concentrations. Slurries increased Cu, Mn, and Zn extractions and plant concentrations of P in straw and Zn in grain. However, the lowest slurry rate was able to maintain the highest grain yields while improving fertility. The results of this research study support the sustainability of pig slurry fertilization at appropriate rates in relation to soil chemical quality.

## Introduction

Pig farming plays an important role in the socio-economic activity of European rural areas, and Spain, with 33 million pigs, is the leading European country in terms of number of animals (Eurostat 2021). Application of pig slurry (PS) to field crops at an agronomic rate is considered a suitable agricultural practice to increase soil quality (Bosch-Serra et al. 2017). If good agricultural practices are applied, environmental impacts such as ammonia volatilization (Bosch-Serra et al. 2014a, b) or greenhouse gas emissions (Shakoor et al. 2021) can be minimized. Pig slurry is also an important source of macronutrients (e.g. N, P, K, Ca, Mg) and micronutrients (e.g. Mn, B, Fe, Ni), especially copper (Cu) and zinc (Zn) (Grohskopf et al. 2016). Pig slurry also contains small amounts of other trace elements that are not nutrients for plants (e.g. Co, Cr, Pb) (Serrano-Barrientos 2001). Copper and Zn are used in feedstuffs to improve animal performance, and eventually for preventing bacterial infections (Suresh et al. 2009; Grohskopf et al. 2016). Digestion and absorption by pigs of these trace elements are limited, and this is why they are transferred to pig slurry (López-Alonso et al. 2012; Kowalski et al. 2013; Montibeller et al. 2017). As a result, regular applications of pig slurry to the soil can raise nutrients and heavy metal levels in both top (0–0.2 m) (Tiecher et al. 2013) and deeper (0.2–0.4 m) soil layers (Veiga et al. 2012). Furthermore, their uptake by plants may also increase (Jakubus et al. 2013; Provolo et al. 2018).

The bioavailability and solubility of micronutrients and other trace elements in the soil profile behave differently when pig slurry is used instead of synthetic fertilizers, because of the complexation of those elements with organic matter (Grohskopf et al. 2016). Mineral fertilization, mainly with P fertilizers, is also a source of additional heavy metals, such as cadmium, according to the fertilizer origin (Mortvedt, 2005). Some of this heavy metals are micronutrients (e.g. Ni, Zn) and others not (e.g. Cr, Pb). In this article, the ‘heavy metal’ (HM) term will be used from selected elements from the heavy metal group that are not nutrients.

Previous research on the effects of pig slurry application on soil HM (Provolo et al., 2018) or on other aspects, such as N efficiency (Bosch-Serra et al. 2015) and N uptake and losses (Ovejero et al. 2016), has been conducted over a relatively low number of cropping seasons. However, research has studied acid or neutral soils (Veiga et al. 2012; Tiecher et al. 2013; Oliveira et al. 2014; Qaswar et al. 2020), irrigated systems (Martínez et al. 2017), or it has developed under greenhouse conditions (Montaghian and Hosseinpur 2015; Provolo et al. 2018). Information about impacts on calcareous soils under field conditions in semiarid rainfed areas is scarce.

Therefore, we hypothesized that fertilization with pig slurry at agronomic rates based on nitrogen (N) criteria sustains soil quality and avoids accumulation of other nutrients and HM. The objective of this research work was to assess, in a semiarid system on calcareous soil, the mid-term effect of pig slurry fertilization (when compared with mineral fertilization) on the folllowing: (1) soil properties (pH, salinity, organic carbon) including soil nutrients (N, P, K, Cu, Zn, Mn, Fe, Ni), and HM concentrations (Co, Cr, Pb); (2) grain and straw biomass of barley (*Hordeum vulgare* L.) in two harvests after 4 and 7 years of pig slurry use; and (3) the concentration of nutrients (N, P, K, Cu, Zn, Mn, Ca, Mg) in barley (in both harvests) after cropping seasons with contrasting rainfall amounts.

## Materials and methods

### Experimental site and study design

A mid-term field experiment (2000–2007) was conducted in Oliola, Lleida, northeastern of Spain. The specific location is 41°52′30″ N, 1°09′1″ E, with an altitude of 440 m a.s.l. The site is located in a slightly sloping (< 2%) valley. No slurry fertilization was applied before the establishment of the experiment. The 2004 sampling was chosen as a midpoint of the experimental period to allow sufficient time since its start for potential studied effects, and to conduct the sampling and analysis of the various variables considered.

Five different treatments with three replications were used during the whole experiment according to a randomized complete block design. The five treatments included a control (C000) without N fertilization, a treatment with mineral (ammonium nitrate) N fertilizer (M090) applied annually at 90 kg N ha^−1^, and three treatments with pig slurry applied annually at 20 m^3^ ha^−1^ (~ 146 kg N ha^−1^) (S146), 40 m^3^ ha^−1^ (~ 281 kg N ha^−1^) (S281) or at 80 m^3^ ha^−1^ (~ 534 kg N ha^−1^) (S534). These amounts cover the range commonly applied by farmers and S146 is the recommended rate. Pig slurry was spread using a conventional splash-plate system before sowing and was incorporated into the soil by disc-harrowing within 24 h of spreading. From 2000 to 2004, annual doses of pig slurry were complemented with 60 kg N ha^−1^ as ammonium nitrate at cereal tillering stage because the experimental field had a very low concentration of organic matter and it had not received any organic fertilizer before the start of the experiment, and therefore, there was no residual N (from organic fertilizers) in the soil profile. Slurries and mineral were always applied in October except in the M090 treatment where 30 kg N ha^−1^ were applied in October and the rest (60 kg N ha^−1^) at cereal tillering stage in February. Phosphorus (P) and potassium (K) were applied annually at sowing in C000 and M090 treatments at 42 kg P ha^−1^ and 89 kg K ha^−1^ in order to avoid shortage of these macronutrients. Therefore, the average amounts of P applied for the S146, S281, and S534 treatments were 26.3 kg ha^−1^, 50.6 kg ha^−1^, and 96.1 kg ha^−1^, respectively. Similarly, the average amounts of K were 122.6 kg ha^−1^, 236.0 kg ha^−1^, and 448.6 kg ha^−1^ for S146, S281, and S534 treatments, respectively. Experimental plots were 12 m long and 7 m wide for C000 and M090 while the rest of the plots were 20 m long and 12 m wide.

Winter cereals were cropped under rainfed conditions following a rotation of 2 years of barley and 1 year of wheat (*Triticum aestivum* L.). Wheat was established in the 2002/2003 and 2005/2006 cropping seasons. The winter cereal was sown in early November, and it was harvested at the end of June. Cereal straw was annually removed from the field and the stubble was buried by tillage before sowing.

Climate in the area is characterized as semiarid Mediterranean, with an average annual rainfall lower than 450 mm and an average reference crop evapotranspiration (ET_o_) of 1013 mm (Penman–Monteith equation; Allen et al. 1998). Daily average air temperature, daily ET_o_, and precipitation data were collected from an automatic weather station next to the experimental field. From 2001 to 2007 annual rainfall ranged from 284 mm (2001) to 593 mm (2003). During the cropping seasons (from October to June) of 2003–2004 and 2006–2007, the accumulated rainfall was 487 mm and 343 mm, respectively. Within years, the maximum monthly rainfall occurred in April, October, or November.

### Sampling and analysis

#### Soil analysis

The soil is a well-drained Typic Xerofluvent (Soil Survey Staff 2014) with over 1 m of rooting depth. Soil samples were collected from 0 to 0.3 m of depth using a soil auger in October 2000 (before any fertilization treatment) and in June 2004 and 2007. Three samples were taken at random in each plot to make a composite sample. Bulk density was measured with the ring method and its average value was 1650 kg m^−3^ in the top layer (0–0.3 m). The content of CaCO_3_ in this layer was 300 g kg^−1^, and the texture was silty loam (USDA), with 609 g kg^−1^ of silt and 260 g kg^−1^ clay. The soil physicochemical properties were determined as follows (Porta et al. 1986): texture by pipette method; pH in aqueous solution using a 1:2.5 (soil:water) ratio and salinity (electrical conductivity, EC) by conductimetry (1:5); total N by Kjeldahl digestion and distillation method (McGill and Figueiredo 1993); oxidizable organic carbon by the Walkley and Black (1934) method; available P content by the Olsen method (sodium bicarbonate-extractable P at pH 8.5; Pansu and Gautheyrou 2003a); cation exchange capacity and exchangeable K which were evaluated by extraction with ammonium acetate 1 N (pH = 7) following Hendershot et al (2008); and further determination by atomic absorption spectrophotometry. A Bernard calcimeter (Pansu and Gautheyrou 2003b) was used for the measurement of calcium carbonate (CaCO_3_). The analysis of total content of micronutrients (Fe, Cu, Mn, Ni, Zn) and the rest of the selected HM (Co, Cr, Pb) was based on the methodology described in EPA 3051 (U.S. EPA 2007) (extraction with 6 mL of 67–69% TraceMetal™ HNO_3_, 2 mL 34–37% TraceMetal™ HCl and 2 mL of Milli-Q water) after microwave digestion. In digested samples, concentrations were determined by inductively coupled mass spectrometry (ICP-MS) in a × 7700 analyzer (Agilent Technologies, Santa Clara, CA, USA), following the UNE-EN 17,053 standard (AENOR 2018). In soil, the micronutrients Cu, Zn, Mn, and Fe were analyzed as they were supplied by slurries (Table 1) and Ni, Co, Cr, and Pb as they were present (traces) in mineral fertilizers, mainly from phosphates (Mortvedt 2005). As C000 received the same amount of P and K fertilizers than M090, micronutrients and the rest of selected heavy metals (not being nutrients) in soil were only analyzed in the C000 treatment.

**Table 1:** Average chemical properties (± standard deviation)^a^ of pig slurry used during the two periods within the whole experimental period

Parameter (units)	First period (*n* = 4) (Oct 2000–Oct 2003)	Second period (*n* = 3) (Oct 2004–Oct 2006)
pH	7.8 ± 0.1	8.6 ± 0.1
EC (dS cm^−1^)	35.7 ± 3.0	25.8 ± 24.3
Dry matter (g kg^−1^ fresh weight)	74 ± 36	102 ± 9
Organic matter (g kg^−1^)	644 ± 71	736 ± 31
Nitrogen (g kg^−1^)	100 ± 36	91 ± 3
Organic N (g kg^−1^)	28 ± 4	27 ± 1
Ammonia N (g kg^−1^)	72 ± 31	64 ± 1
P (g kg^−1^)	20 ± 0	14 ± 0
K (g kg^−1^)	83 ± 40	77 ± 16
Ca (g kg^−1^)	26 ± 6	34 ± 17
Mg (g kg^−1^)	9 ± 3	10 ± 0
Na (g kg^−1^)	19 ± 7	14 ± 1
Fe (mg kg^− 1^)	3871.7 ± 161.4	2958.3 ± 271.0
Mn (mg kg^−1^)	492.0 ± 127.0	624.4 ± 150.2
Cu (mg kg^−1^)	751.6 ± 183.4	361.5 ± 133.6
Zn (mg kg^−1^)	1661.1 ± 678.0	1330.7 ± 265.9

^a^It represents the average and standard deviation of the applied pig slurry for each period. Values refer to dry weight

#### Pig slurry collection and analysis

Pig slurry was always collected from a nearby fattening pig farm and slurry samples were collected at application time, just before annual sowing. Slurries were analyzed for dry matter after drying at 105 °C, pH and electrical conductivity (EC) in a 1:5 dilution in water, organic matter as loss of weight after calcination at 540 °C, total and ammonia N by the Kjeldahl method, and P, K, Ca, Mg, Na, Fe, Mn, Cu, and Zn by ICP-MS spectrometry following the EPA 3051 procedure (U.S. EPA 2007) (Table 1). The amounts applied of all these elements and sodium (Na) were calculated for the periods leading to the two harvests analyzed, from October 2000 to October 2003 and from October 2004 to October 2006 (Table 2). In the last cropping season, the amount of N applied with slurries (October 2006) was 99, 211, and 402 kg N ha^−1^ for treatments S146, S281, and S534, respectively, because of the variability in slurry composition.

**Table 2:** Organic carbon, nutrients, and Na applied in the different treatments^a^ and in two periods^b^: from October 2000 to October 2003 (period 1, four fertilizer applications) and from October 2004 to October 2006 (period 2, three fertilizer applications)

Treatment	C000	M090	S146	S281	S534
**Accumulated organic carbon applied (kg ha**^**−1**^**)**					
Period 1	—	—	2469.5	4318.5	8285.4
Period 2	—	—	1544.6	3174.1	6285.0
**Total**	—	—	4014.1	7492.6	14,570.4
**Accumulated N applied (kg ha**^**−1**^**)**					
Period 1^**c**^	0	360	513.9	1016.2	1913.2
Period 2	0	270	284.2	625.8	1215.0
**Total**	0	630	798.1	1642.0	3128.2
**Accumulated P applied (kg ha**^**−1**^**)**					
Period 1	169	169	116.9	231.0	433.0
Period 2	126	126	44.3	98.1	200.0
**Total**	295	295	161.2	329.1	633.0
**Accumulated K applied (kg ha**^**−1**^**)**					
Period 1	357	357	388.5	855.7	1515.3
Period 2	268	268	234.7	518.5	1107.8
**Total**	625	625	623.2	1374.2	2623.1
**Accumulated Ca applied (kg ha**^**−1**^**)**					
Period 1	—	—	172.7	305.9	605.1
Period 2	—	—	107.3	240.0	503.2
**Total**	—	—	280.0	545.9	1108.3
**Accumulated Mg applied (kg ha**^**−1**^**)**					
Period 1	—	—	64.1	116.8	232.8
Period 2	—	—	32.0	70.9	144.0
**Total**	—	—	96.1	187.7	376.8
**Accumulated Na applied (kg ha**^**−1**^**)**					
Period 1	—	—	99.1	213.5	372.3
Period 2	—	—	43.5	93.2	195.8
**Total**	—	—	142.6	306.7	568.1
**Accumulated Fe applied (kg ha**^**−1**^**)**					
Period 1	—	—	25.5	51.1	98.6
Period 2	—	—	8.9	19.9	40.1
**Total**	—	—	34.4	71.0	138.7
**Accumulated Mn applied (kg ha**^**−1**^**)**					
Period 1	—	—	3.1	5.7	11.3
Period 2	—	—	1.9	6.9	8.8
**Total**	—	—	5.0	12.6	20.1
**Accumulated Cu applied (kg ha**^**−1**^**)**					
Period 1	—	—	4.6	9.6	17.7
Period 2	—	—	1.0	2.3	4.7
**Total**	—	—	5.6	11.9	22.4
**Accumulated Zn applied (kg ha**^**−1**^**)**					
Period 1	—	—	10.8	22.4	41.3
Period 2	—	—	3.9	8.7	18.0
**Total**	—	—	14.7	31.1	59.3

^a^The letter in the acronym indicates the fertilizer origin: mineral fertilizer (M), pig slurry (S), and the control (C). The numbers indicate the average of N rate applied annually as slurry or only mineral (kg ha^−1^)

^b^Slurries and mineral were always applied in October except in the M090 treatment where 30 kg N ha^−1^ were applied in October and the rest (60 kg N ha^−1^) at cereal tillering stage in February

^c^In the first period of time, slurry treatments received at cereal tillering a complementary amount of 60 kg N ha^−1^ as ammonium nitrate, which means a complementary addition of 240 kg N ha^−1^ in period 1 which is not included in the figures of the table

#### Plant analysis

Barley plant samples were taken at the 2004 and 2007 harvests. Straw and grain samples were analyzed for N according to the Kjeldahl method. Other samples (0.25 g) were digested according to EPA 3052 methodology (U.S. EPA 1996), with a mixture of 4 mL 67–69% TraceMetal™ HNO_3_, 2 mL of H_2_O_2_ at 30% and 4 mL of Milli-Q water. Concentrations of other nutrients were quantified following the previously described ICP-MS method. Plant uptake was estimated by multiplying straw and grain biomass by their element or nutrient concentration. In plants, macronutrients (N, P, K, Ca, and Mg) and the micronutrients Cu, Zn, and Mn were analyzed.

#### Statistical analysis

The data was statistically analyzed by using the statistical package SAS (v 9.4) (SAS Institute 2014). Analysis was performed by the maximum likelihood method to analyze the effects of treatments and sampling year on soil chemical variables and on element concentrations and contents in the different fractions (grain and straw) of aerial crop biomass. The SAS System’s MIXED procedure (Littell et al 1996) was used for all performed analyses. Basic statistical assumptions were checked. Treatments and years were considered as fixed effects and replications as random effect. We selected a value of 5% (i.e., *p* < 0.05) as the minimum criterion for significance. The standard error of differences (SED) and the least significant difference (LSD) were calculated according to Webster (2007) and Webster and Lark (2018).

## Results

### Grain and straw yields

**Fig. 1:**
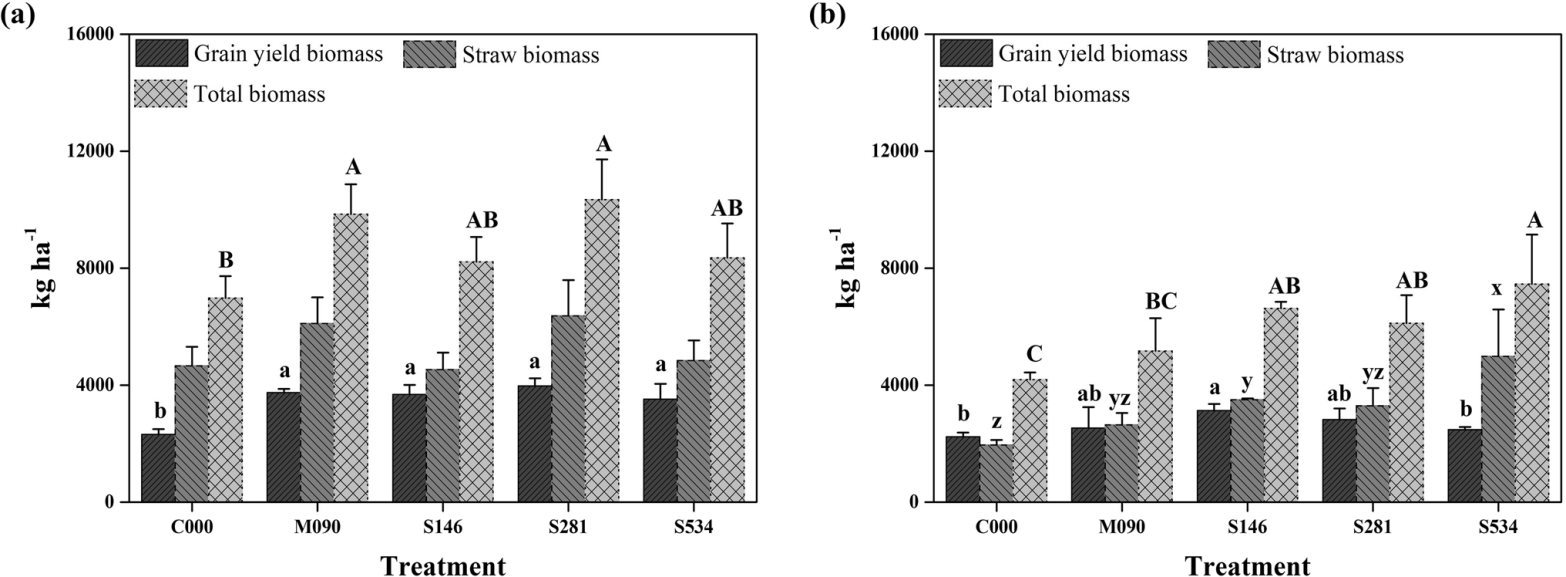
Average grain yield, straw, and total biomass according to pig slurry treatments maintained for **a** 2004 and **b** 2007 cropping seasons. Vertical bars indicate ± one standard deviation of the mean (*n* = 3). Significant differences between means are showed by different letters and according to LSD test; lowercase letters were used for grain (a, b) and straw (x, y, z) biomass and uppercase letters for total biomass

In 2004, the control had lower grain yield (2312 kg ha^−1^) than the rest of the treatments (average of 3724 kg ha^−1^), but no significant differences appeared in straw biomass (Fig. 1a). In 2007 (Fig. 1b), the grain yield of the S146 treatment (3125 kg ha^−1^) was higher than that of the S534 treatment (2474 kg ha^−1^) although S534 produced the highest straw biomass (4982 kg ha^−1^). The control treatment also yielded lower grain and straw biomass than S146 in this year.

### Pig slurry effects on soil properties and fertility

In the experimental period of 7 years, no significant changes appeared in soil pH or cation-exchange capacity (CEC) (Table 3), with mean values of 8.2 and 8.4 cmol^+^ kg^−1^, respectively. All treatments tended to increase soil EC 1:5 with time (from 0.2 up to 0.3 dS m^−1^ at 25 °C). Soil organic carbon (SOC) concentration only increased significantly (by an average of 20%) when the control was compared with the two highest slurry rates of S281 and S534 (Table 3). With the exception of the control, total soil N (Kjeldahl N) also increased with time in all treatments. In 2007, it also increased with N rate but only the S281 and S534 treatments (with an increase of 16% and 33%, respectively) were significantly different from the control (Table 3). The similar SOC and total N changes led to a constant C:N ratio average, around 9.1–9.2. Available Olsen P significantly increased, from an initial average of 10.7 mg kg^−1^ in 2000, at a rate of 3.1 mg P kg ^−1^ soil for every 100 kg P ha^−1^ in S146, and at rate 4.6 mg P kg ^−1^ soil for higher doses (Tables 2 and 3). Thus, a maximum concentration of 41.0 mg kg^−1^ was recorded for the S534 treatment. The highest rates of slurry also produced significant increases in soil total P compared to S146. The relation between available P increase and total P increase was 12%, 10%, and 17% for S146, S281, and S534, respectively. For the control, this relation was 10%. Available K increased significantly from an average of 96.1 mg K kg^−1^ in 2000 to 209.1 mg K kg^−1^ in 2007, with a highest value of 302 mg K kg^−1^ in the S534 treatment (Table 3). For micronutrients and HM, no interactions were found. Differences between years were also detected in total Cu and Zn soil concentrations (Table 4). No differences in total Mn, Fe, Ni, Co, and Cr soil concentrations were detected (Table 4) with time or between treatments.

**Table 3:** Values^a^ of physicochemical properties of soil and its macronutrient concentrations when the experiment was established (year 2000) and after 7 years (2007) according to annual pig slurry treatments applied during this period (2000–2007). Marginal means for years (*MM*_year_) and treatments (*MM*_treat_) are also included

Parameter	Treatment	2000	2007	*MM*_treatments_
pH	C000	8.3	8.1	8.2 ± 0.05
	M090	8.2	8.2	8.2 ± 0.05
	S146	8.1	8.2	8.2 ± 0.05
	S281	8.3	8.2	8.3 ± 0.05
	S534	8.3	8.2	8.2 ± 0.05
	*MM*_year_	8.2 ± 0.03	8.2 ± 0.03	
Soil EC 1:5 (dS m^−1^, 25 °C)	C000	0.2	0.3	0.2 ± 0.02
	M090	0.2	0.3	0.2 ± 0.02
	S146	0.2	0.3	0.2 ± 0.02
	S281	0.2	0.2	0.2 ± 0.02
	S534	0.2	0.3	0.2 ± 0.02
	*MM*_year_	0.2 ± 0.01 Y	0.3 ± 0.01 X	
Soil CEC (cmol^+^ kg^−1^)	C000	7.8	7.9	7.9 ± 0.68
	M090	-	-	-
	S146	7.7	8.8	8.2 ± 0.68
	S281	9.1	9.1	9.1 ± 0.68
	S534	8.5	7.7	8.1 ± 0.68
	*MM*_year_	8.3 ± 0.54	8.4 ± 0.54	
Soil OC (g kg^−1^)	C000	10.0	11.0	10.5 ± 0.49 B
	M090	10.0	12.0	11.0 ± 0.49 AB
	S146	10.0	12.0	11.0 ± 0.49 AB
	S281	11.0	13.0	12.0 ± 0.49 A
	S534	10.0	14.0	12.0 ± 0.49 A
	*MM*_year_	10.1 ± 0.38 Y	11.6 ± 0.38 X	
N (Kjeldahl method) (g kg^−1)^	C000	1.1 a x	1.2 c x	1.1 ± 0.05
	M090	1.1 a y	1.3 bc x	1.2 ± 0.05
	S146	1.1 a y	1.3 bc x	1.2 ± 0.05
	S281	1.1 a y	1.4 ab x	1.2 ± 0.05
	S534	1.0 a y	1.6 a x	1.3 ± 0.05
	*MM*_year_	1.0 ± 0.04	1.3 ± 0.04	
C:N ratio	C000	9.4	9.1	9.3 ± 0.36
	M090	9.5	9.3	9.4 ± 0.36
	S146	8.9	9.0	9.0 ± 0.36
	S281	9.3	9.2	9.3 ± 0.36
	S534	9.8	8.8	9.3 ± 0.36
	*MM*_year_	9.2 ± 0.27	9.1 ± 0.27	
Available P (Olsen) (mg kg^−1^)	C000	11.0 a	35.67 ab	23.33 ± 1.94
	M090	10.3 a	17.67 c	14.00 ± 1.94
	S146	9.7 a	14.7 c	12.2 ± 1.9
	S281	11.3 a	26.0 bc	18.7 ± 1.94
	S534	11.3 a	41.0 a	26.2 ± 1.94
	*MM*_year_	10.7 ± 1.23 Y	27.0 ± 1.23 X	
Available K (mg kg^−1^)	C000	81.0 a	205.0 b	143.0 ± 12.45
	M090	102.3 a	178.0 bc	140.2 ± 12.45
	S146	94.7 a	154.7 c	124.7 ± 12.45
	S281	104.3 a	205.7 b	155.0 ± 12.45
	S534	98.3 a	302.0 a	200.2 ± 12.45
	*MM*_year_	96.1 ± 8.43 Y	209.1 ± 8.43 X	
Total P (mg kg^−1^)	C000	480.7	719.7	600.2 ± 32.23 AB
	M090	-	-	-
	S146	566.3	607.3	586.8 ± 32.23 B
	S281	621.0	770.3	695.7 ± 32.23 A
	S534	607.3	779.7	693.5 ± 32.23 A
	*MM*_year_	568.8 ± 22.79 Y	719.3 ± 22.79 X	

^a^Means followed by a different letter are significantly different according to the least significant difference, all for *P* = 0.05. Capital letters X and Y are used for differences between years and the capital letters A and B are used for differences between treatments. When an interaction was found, lower case letters x and y are used to differentiate between years (for each treatment), and lower case letters a, b, and c are used to differentiate between treatments (for each year)

**Table 4:** Micronutrient and soil heavy metal contents^a^ after 4 years (2004 sampling) from the experiment establishment (year 2000) and 7 years later (2007), and according to different annual pig slurry treatments applied during this period (2000–2007). Marginal means for years (*MM*_year_) and treatments (*MM*_treat_) are also included

Parameter	Treatment	2000	2007	*MM*_treatments_
Co (mg kg^−1^)	C000	8.1	8.5	8.3 ± 0.34
	S146	8.8	8.8	8.8 ± 0.34
	S281	8.9	8.9	8.9 ± 0.34
	S534	8.4	8.4	8.4 ± 0.34
	*MM*_year_	8.5 ± 0.27	8.6 ± 0.27	
Cr (mg kg^−1^)	C000	17.7	12.7	15.2 ± 1.07
	S146	15.0	14.7	14.8 ± 1.07
	S281	15.0	17.3	16.2 ± 1.07
	S534	13.7	14.3	14.0 ± 1.07
	*MM*_year_	15.3 ± 0.71	14.8 ± 0.71	
Cu (mg kg^−1^)	C000	20.0	20.0	20.0 ± 1.31
	S146	19.7	23.0	21.3 ± 1.31
	S281	16.0	22.7	19.3 ± 1.31
	S534	17.0	24.7	20.8 ± 1.31
	*MM*_year_	18.2 ± 1.03 Y	22.6 ± 1.03 X	
Fe (mg kg^−1^)	C000	20,987.4	21,486.1	21,236.7 ± 604.23
	S146	22,112.9	22,065.6	22,089.2 ± 604.23
	S281	22,573.0	22,671.7	22,622.3 ± 604.23
	S534	21,577.2	21,217.3	21,397.3 ± 604.23
	*MM*_year_	21,813 ± 461.2	21,860 ± 461.2	
Mn (mg kg^−1^)	C000	564.3	588.0	576.2 ± 24.27
	S146	604.3	626.7	615.5 ± 24.27
	S281	598.3	596.3	597.3 ± 24.27
	S534	568.3	547.7	558.0 ± 24.27
	*MM*_year_	583.8 ± 21.75	588.7 ± 21.75	
Ni (mg kg^−1^)	C000	21.3	23.3	22.3 ± 1.06
	S146	23.3	24.0	23.7 ± 1.06
	S281	24.0	24.7	24.3 ± 1.06
	S534	22.7	23.0	22.8 ± 1.06
	*MM*_year_	22.8 ± 0.91	23.7 ± 0.91	
Pb (mg kg^−1^)	C000	18.7	18.7	18.7 ± 0.81
	S146	19.0	19.7	19.3 ± 0.81
	S281	19.3	19.7	19.5 ± 0.81
	S534	18.3	19.0	18.7 ± 0.81
	*MM*_year_	18.8 ± 0.67	19.3 ± 0.67	
Zn (mg kg^−1^)	C000	61.0	67.3	64.2 ± 3.68 B
	S146	68.7	74.3	71.5 ± 3.68 AB
	S281	70.7	85.0	77.8 ± 3.68 A
	S534	67.0	89.7	78.3 ± 3.68 A
	*MM*_year_	66.8 ± 2.86 Y	79.1 ± 2.86 X	

^a^Means followed by a different letter are significantly different according to the least significant difference, all for *P* = 0.05. Capital letters X and Y are used for differences between years and the capital letters A and B are used for differences between treatments

### Pig slurry effects on element concentrations and uptake in plants

The highest concentrations of N and P in grain were reached with the highest slurry dose (Table 5), while the rest of the slurry treatments gave results similar to those of the mineral treatment. From 2004 to 2007, grain concentrations of N, Mg, Cu, and Mn increased and the opposite was observed for P and K (Table 5). Only treatments S281 and S534 resulted in an increase between years in Zn grain concentration; in 2007, both treatments also showed higher Zn concentrations than the control.

**Table 5:** Nutrient concentrations^a^ in barley (*Hordeum vulgare* L.) grain in 2004 and 2007 harvests and according to different annual treatments in an experiment established in the 2000–2001 cropping season. Marginal means for years (*MM*_year_) and treatments (*MM*_treat_) are also included

Parameter	Treatment	2000	2007	*MM*_treatments_
N (g kg^−1^)	C000	14.9	23.0	19.0 ± 1.11 C
	M090	16.9	31.4	24.1 ± 1.11 B
	S146	17.4	28.3	22.9 ± 1.11 B
	S281	19.3	32.0	25.7 ± 1.11 B
	S534	21.2	36.9	29.1 ± 1.11 A
	*MM*_year_	17.9 ± 0.80 Y	30.3 ± 0.80 X	
P (g kg^−1^)	C000	4.2	3.5	3.8 ± 0.11 C
	M090	4.1	3.9	4.0 ± 0.11 BC
	S146	4.3	3.7	4.0 ± 0.1 BC
	S281	4.3	4.0	4.1 ± 0.11 B
	S534	4.5	4.7	4.6 ± 0.11 A
	*MM*_year_	4.2 ± 0.07 X	3.9 ± 0.07 Y	
K (g kg^−1^)	C000	5.6	4.2	4.9 ± 0.10
	M090	5.3	4.6	4.9 ± 0.10
	S146	5.5	4.3	4.9 ± 0.10
	S281	5.7	4.3	5.0 ± 0.10
	S534	5.3	4.5	4.9 ± 0.10
	*MM*_year_	5.4 ± 0.07 X	4.3 ± 0.07 Y	
Ca (g kg^−1^)	C000	0.6	0.4	0.5 ± 0.04
	M090	0.5	0.6	0.5 ± 0.04
	S146	0.5	0.5	0.5 ± 0.04
	S281	0.6	0.6	0.5 ± 0.04
	S534	0.6	0.7	0.6 ± 0.04
	*MM*_year_	0.5 ± 0.03	0.5 ± 0.03	
Mg (g kg^−1^)	C000	1.2	1.2	1.2 ± 0.04
	M090	1.1	1.3	1.2 ± 0.04
	S146	1.2	1.3	1.2 ± 0.04
	S281	1.1	1.3	1.2 ± 0.04
	S534	1.1	1.4	1.2 ± 0.04
	*MM*_year_	1.1 ± 0.02 Y	1.3 ± 0.03 X	
Cu (mg kg^−1^)	C000	2.6	3.7	3.3 ± 0.52
	M090	2.5	5.0	3.7 ± 0.47
	S146	2.3	5.3	3.8 ± 0.47
	S281	4.7	5.3	5.0 ± 0.48
	S534	3.8	6.3	5.0 ± 0.52
	*MM*_year_	3.2 ± 0.29 Y	5.1 ± 0.26 X	
Mn (mg kg^−1^)	C000	17.9	18.3	18.1 ± 0.62
	M090	18.1	19.7	18.9 ± 0.62
	S146	19.9	19.0	19.4 ± 0.62
	S281	16.8	20.7	18.7 ± 0.62
	S534	18.2	22.0	20.1 ± 0.62
	*MM*_year_	18.2 ± 0.39 Y	20.0 ± 0.39 X	
Zn (mg kg^−1^)	C000	25.2 b x	23.7 c x	24.4 ± 2.11
	M090	23.4 b y	30.0 bc x	26.7 ± 2.11
	S146	29.7 a x	29.7 bc x	29.8 ± 2.11
	S281	23.5 b y	35.0 ab x	29.2 ± 2.11
	S534	27.1 ab y	41.3 a x	34.2 ± 2.29
	*MM*_year_	25.8 ± 1.23	31.9 ± 1.18	

^a^Means followed by a different letter are significantly different according to the least significant difference, all for *P* = 0.05. Capital letters X and Y are used for differences between years and the capital letters A and B are used for differences between treatments. When an interaction was found, lower case letters x and y are used to differentiate between years (for each treatment), and lower case letters a, b, and c are used to differentiate between treatments (for each year)

In barley straw (Table 6), N concentration increased with time in all treatments with the sole exception of the control. The control always showed lower N concentration than S281 and S534. The highest slurry dose also attained the highest P straw concentration while S281 led to the lowest Ca concentration. The Ca, Zn, and Cu concentrations increased with time. No differences were observed for K, Mg, and Mn concentrations.

**Table 6:** Nutrient concentrations^a^ in barley (*Hordeum vulgare* L.) straw in 2004 and 2007 harvests and according to different annual mineral and pig slurry treatments in an experiment established in the 2000–2001 cropping season. Marginal means for years (*MM*_year_) and treatments (*MM*_treat_) are also included

Parameter	Treatment	2000	2007	*MM*_treatments_
pH	C000	8.3	8.1	8.2 ± 0.05
	M090	5.1 ab y	7.7 bc x	6.4 ± 0.60
	S146	4.7 b y	6.4 bc x	5.5 ± 0.60
	S281	6.8 a y	8.7 b x	7.8 ± 0.60
	S534	7.0 a y	15.2 a x	11.1 ± 0.60
	*MM*_year_	5.6 ± 0.40	8.5 ± 0.40	
P (g kg^−1^)	C000	1.1	0.5	0.6 ± 0.11 C
	M090	0.8	0.8	1.0 ± 0.11 B
	S146	0.8	0.6	0.7 ± 0.11 BC
	S281	0.9	0.8	0.9 ± 0.11 BC
	S534	1.2	1.4	1.3 ± 0.11 A
	*MM*_year_	1.1 ± 0.07	0.8 ± 0.07	
K (g kg^−1^)	C000	14.2	13.5	13.8 ± 1.40
	M090	18.7	17.4	18.1 ± 1.40
	S146	15.7	14.2	15.0 ± 1.40
	S281	14.7	17.7	16.2 ± 1.40
	S534	20.5	20.4	20.4 ± 1.40
	*MM*_year_	16.7 ± 0.80	16.6 ± 0.80	
Ca (g kg^−1^)	C000	6.7	5.4	6.1 ± 0.3 AB
	M090	7.3	6.4	6.8 ± 0.30 A
	S146	6.8	5.8	6.4 ± 0.30 A
	S281	4.9	5.5	5.2 ± 0.30 B
	S534	7.4	6.1	6.7 ± 0.30 A
	*MM*_year_	6.6 ± 0.20 X	5.8 ± 0.20 Y	
Mg (g kg^−1^)	C000	1.1	0.8	1.0 ± 0.12
	M090	1.2	1.0	1.0 ± 0.13
	S146	1.0	0.7	0.8 ± 0.12
	S281	0.9	1.1	1.0 ± 0.12
	S534	1.0	0.7	0.8 ± 0.12
	*MM*_year_	1.0 ± 0.09	0.8 ± 0.08	
Cu (mg kg^−1^)	C000	3.4	4.7	4.1 ± 0.55
	M090	4.0	5.3	4.7 ± 0.49
	S146	3.9	5.3	4.6 ± 0.55
	S281	3.9	6.7	5.2 ± 0.49
	S534	5.4	6.3	5.8 ± 0.49
	*MM*_year_	4.1 ± 0.34 Y	5.7 ± 0.31 X	
Mn (mg kg^−1^)	C000	39.6	32.0	35.8 ± 6.90
	M090	32.6	37.0	34.8 ± 7.55
	S146	42.7	52.0	35.3 ± 6.90
	S281	42.6	51.6	48.3 ± 6.90
	S534	53.7	60.0	39.5 ± 6.90
	*MM*_year_	42.2 ± 4.76	35.2 ± 4.60	
Zn (mg kg^−1^)	C000	5.8	7.5	6.6 ± 0.21
	M090	5.4	8.6	7.0 ± 0.32
	S146	8.9	8.7	8.8 ± 0.42
	S281	7.0	11.0	9.0 ± 0.55
	S534	10.7	17.0	13.9 ± 0.69
	*MM*_year_	7.56 ± 0.65 Y	10.6 ± 0.31 X	

^a^Means followed by a different letter are significantly different according to the least significant difference, all for *P* = 0.05. Capital letters X and Y are used for differences between years and the capital letters A and B are used for differences between treatments. When an interaction was found, lower case letters x and y are used to differentiate between years (for each treatment), and lower case letters a, b, and c are used to differentiate between treatments (for each year)

**Fig. 2:**
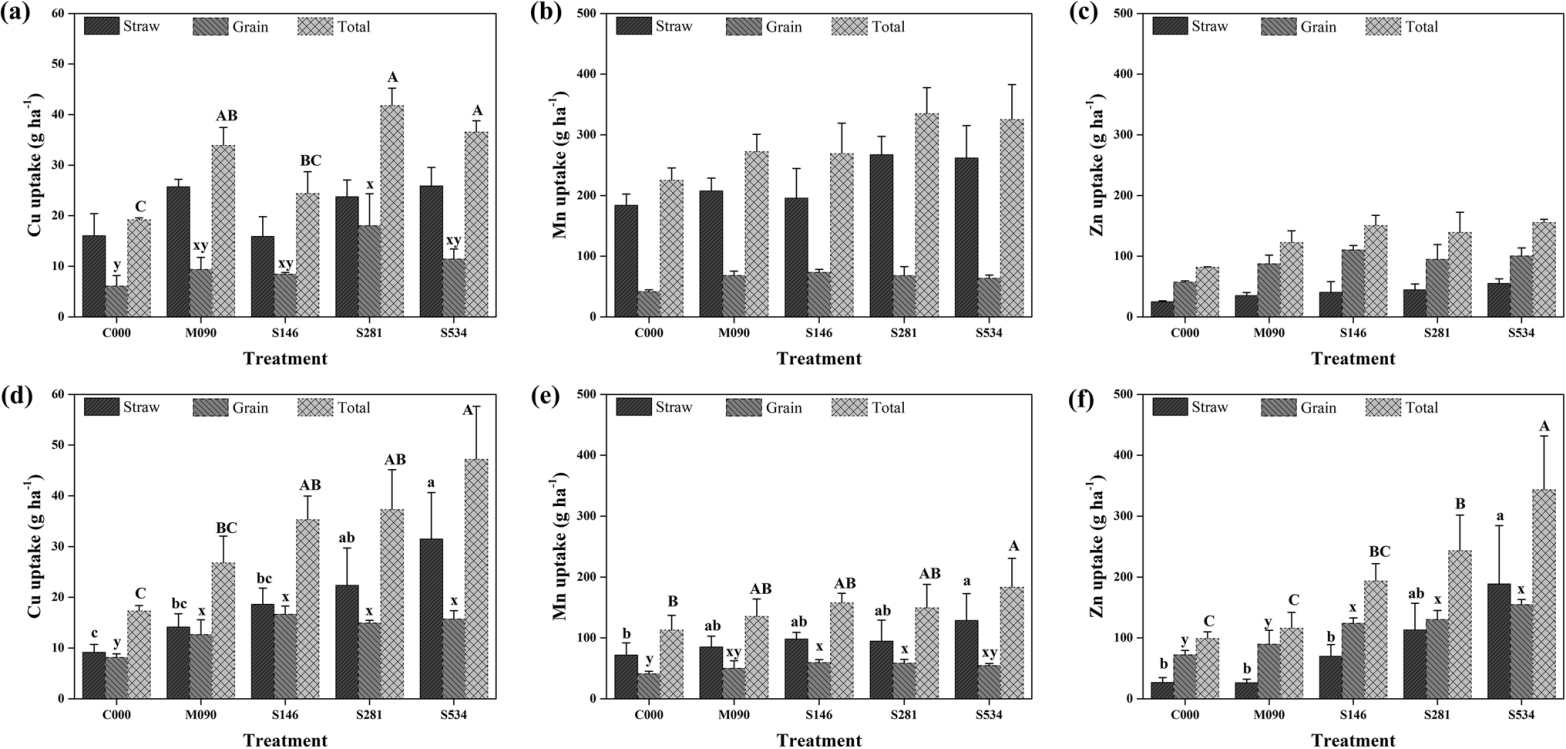
Copper, manganese, and zinc average uptakes by straw, grain, and total biomass in 2004 (**a**, **b**, **c**) and 2007 (**d**, **e**, **f**) cropping seasons according to different annual pig slurry treatments. Vertical bars indicate ± one standard deviation of the mean (*n* = 3). Significant differences between means are showed by different letters and according to LSD test; lowercase letters were used for straw (a, b, c) and grain (x, y) biomass and uppercase letters for total biomass

Differences in element concentration and plant biomass led to significant differences in Cu and Mn plant uptake between treatments in both harvests and to differences in Zn uptake in the 2007 harvest (Fig. 2). No significant differences in uptake were obtained for the other elements.

## Discussion

### Soil properties

In this experiment, the high soil CaCO_3_ content (300 g kg^−1^) prevented any acidification of the soil even at the higher rates of pig slurry applications. Changes in EC 1:5 were also non-significant between treatments (Table 3) and changes between sampling periods were likely due to a dry period prior to sampling in 2007.

Soil organic carbon concentration at the beginning of the experiment was close to 10 g kg^−1^, which is considered the lower limit for sustainability (Jones et al. 2004), but all the fertilization treatments increased SOC with time (Table 3) despite the removal of straw. However, differences were only significant between the highest doses of pig slurry and the control, probably because of the low organic matter concentration of the slurry applied (Table 1). Nevertheless, it has been found that fresh organic matter from pig slurry helps to improve physical conditions (Oliveira et al. 2014) as well microbial activity (Valdez et al. 2020) which may justify the higher total plant biomass in S534 than in M090 in 2007 (Fig. 1b), despite the excessive amount of N applied.

The N remaining in the soil profile at the end of the cropping season may benefit subsequent crops, which is known as the residual effect (Albuquerque et al. 2017), but losses to soil and air should remain under certain acceptable limits. In the last harvest of 2007, the ratio between N yield of M090 vs. the one of the S146 treatment (Fig. 1b, Table 5) was 1.1 but the lower ratio of other treatments close to 0.4 led to the rest of slurry treatments not being considered to give sustainable fertilization approaches. Also, the ratio of N output/ N input (in this case at plot level) and according to the EU Nitrogent Expert Panel (2015) should achieve a minimum of 0.5 to be considered sustainable. Again, only treatment S146 may be considered sustainable under this criterion, because it reaches a value of 0.8 in 2007 vs. the value of 0.4 for S281 and S534 (Fig. 1b, Tables 5 and 6).

It has also been reported that pig slurry may increase soil productivity, above and over its nutrient contents, when large inputs are applied to soil over several years (Edmeades 2003). Results from this study demonstrate that the yearly application of pig slurry significantly increased soil fertility in the mid-term (Table 3), which agrees with the results obtained working with manures (Mahmood et al. 2017). At the beginning of the experiment, available P and K (Table 3) concentrations were low for a silty loam soil (Rodríguez Martín et al. 2009). After a 7-year period, the increment in these concentrations might be explained by the higher application rates of pig slurry or minerals in comparison to the exportations from the crops (grain and straw) (Fig. 1, Table 6). In the S146 treatment, P and K extractions, in 2007 and in 2004, were 61–88% (P) and 79–100% (K) of the amounts applied in those years. It must be stressed that in 2004, yields were higher than in 2007. The N agronomic treatment (S146) improved fertility but K remained at acceptable levels according to the CEC of the soil (Cottenie 1980); for the rest of treatments, the attained concentrations were high (M090) or very high (over 200 mg K kg^−1^).

Olsen P concentrations above 35–40 mg kg^−1^ in the plough layer are considered critical points in terms of risk of P leaching (Hughes et al. 2000; Bai et al. 2013). Although the two highest pig slurry treatments reported in this paper showed large increases in Olsen P concentration, critical levels were only reached with the highest one. This fact reinforces the choice of the S146 treatment as the recommendable rate in ths rainfed winter cereal system. It must be pointed out that in nitrate vulnerable zones (Generalitat de Catalunya 2019), in order to protect water quality and according to EU regulations, the maximum amount of N from organic origin to be applied is 170 kg N ha^−1^. This dose matches our findings, although the experimental area is not included in an existing vulnerable area. Recommendations by the Common Agricultural Policy of the European Union to include leguminous crops in the rotation cycle should also help to control P levels because they can make a significant use of the P in soils.

From 2004 to 2007, the application of slurries increased soil concentrations around 1.3 mg Zn kg^−1^ soil for every 1 kg Zn ha^−1^ applied, and 0.4 mg Cu kg^−1^ soil for every 1 kg Cu ha^−1^ applied (Tables 2 and 4), although the amount (kg ha^−1^) of Cu applied (from 2004 to 2007) was around four times less than that of Zn (Table 2). These increases are consistent with the results obtained by Kumaragamage et al. (2016), and the mean concentrations of 79.1 mg Zn kg^−1^ and 22.6 mg Cu kg^−1^ obtained in 2007 are within the ranges proposed by Barber (1995) (10–300 mg kg^−1^ for Zn and 1–50 mg kg^−1^ for Cu). The total increases are the result of the adsorption of these elements in soils at high pH into positions from which they are not readily displaceable. The absence of differences for other elements such as Mn and Fe may be due to the low amounts applied (Table 2) compared with the soil contents (Table 4).

In relation to heavy metals, the final average soil concentrations of Cr (14.8 mg kg^−1^), Ni (23.7 mg kg^−1^), and Pb (19.3 mg kg^−1^) are far below the thresholds (150 mg Cr kg^−1^, 112 mg Ni kg^−1^, 300 mg Pb kg^−1^) established by the Spanish government for basic soils (pH > 7) receiving urban sludge (MAPA 1990), according to EU regulations. The cobalt concentration in soil of 8.4 mg kg^−1^ obtained in 2007 is considered to be within the modal range for soils (Page et al 1981).

### Crop yield and element concentrations

Rainfall differences, 100 mm less in the 2006–2007 than in the 2003–2004 cropping season, favoured grain yield in S146 as its green biomass (leaves and stem) was smaller than for S534. This fact may have reduced grain filling water stress at the end of the growing season due to a lower evaporative demand in S146 than in S534. Thus, we may consider the 146 kg N ha^−1^ pig slurry dose as the long-term advisable agronomic treatment. Its performance also compares very well with that of a mineral fertilization of 90 kg N ha^−1^ (Fig. 1).

In plants, changes in N concentration between harvests can be explained by the dilution effect (Greenwood and Draycott 1989), which means that as yields increase (as was the case in 2004 vs. 2007), N concentration diminishes (Table 5). Furthermore, if N application rates increase, N concentration can increase, which is the case with the S534 treatment, which shows the maximum concentrations in grain and straw (Tables 5 and 6).

The highest slurry dose, which also involved the highest P application rate, produced the highest concentrations of P both in grain and straw, probably as a result of higher P concentration in the soil solution. Higher rainfall may also explain the significantly higher concentration of P in grain in 2004 compared to 2007as P availability for plants was increased because P is transported in the soil mainly through the diffusion process (Olsen and Watanabe 1963).

Lack of water could also constrain K grain availability in 2007, as the reduction in volumetric water content reduced K diffusion in soil due to a reduction in cross-sectional area for diffusion and an increment in the tortuosity of the diffusion path (Barber 1995). Calcium is fully available in this calcareous soil, and therefore, the lowest value in barley straw in 2004 and in S281 treatment could not be related to Ca availability. The higher Mg grain concentration in 2007 vs. 2004 may be also explained by the dilution effect in the latter year.

Copper concentrations in barley (Tables 5 and 6) were within the lowest range of normal Cu concentrations (from 3–20 mg kg^−1^ of dry foliage) according to Chaney (1989) which means that slurries were not able to improve plant Cu availability and differences with time may be attributed to a dilution effect similar to that found for other elements. According to Montaghian and Hosseinpur (2015), in calcareous soils amended with sewage sludges, there is a redistribution of Cu between different soil fractions, increasing the proportion linked to organic matter. However, final Cu extractions by barley plants increased with slurry fertilization (Fig. 2d) which means that the slurry had a positive effect on Cu bioavailability, as has also been found by Zaragüeta et al. (2021) in a calcareous soil fertilized with sewage sludge.

In the case of Mn, the grain concentration increased in 2007 in comparison to 2004, which may also be related to the dilution effect caused by yield differences between years, as has been previously found by Morera et al. (2002). Concentrations of Mn (Tables 5 and 6) were in the adequacy range of 20–500 mg kg^−1^ (Anderson and Ohki 1977). However, plants were able to positively answer to fertilization, as extraction increased when compared to the control, mainly due to a higher Mn straw extraction (Figs. 2b, e). This response to Mn applications (Table 2) has been previously recorded in calcareous soils (Reuter et al. 1973), even though solubility of Mn compounds is reduced in basic soils.

The Zn concentrations in plants in our experiment (Table 5) were within the lower range of values for most crops and pasture plants (10 to 100 mg kg^−1^) (Lindsay 1972). Nevertheless, slurry fertilization increased these concentrations, and therefore grain quality, and the highest Zn concentration in grain coincided with the highest slurry rate (Table 5) which also allowed, in 2007 the highest extraction. Therefore, slurry was necessary to improve Zn fertility levels and increase Zn plant extraction (Fig. 2f).

The previous results are in agreement with Provolo et al. (2018) who found that soils with a long history of pig slurry application were associated with higher contents of Mn, Cu, and Zn in plant shoots.

## Conclusions

In this rainfed semiarid system, an average pig slurry dose of 146 kg N ha^−1^ produced barley grain yields (~ 3–4 Mg ha^−1^) as high as those obtained with mineral fertilization or with doses with a higher N content. Such dose also increased soil organic carbon, total N, available P and K, and total Cu and Zn to levels within acceptable fertility ranges, and did not significantly affect heavy metal concentrations. Slurry application affected N, P, and Zn concentrations in grain and N and P concentration in straw, but nutrient concentrations in plants also varied with accumulated rainfall during the cropping season: its reduction increased N, Mg, Cu, Zn, and Mn concentrations in barley grain and reduced those of K and P, while N, Cu, and Zn concentrations in straw increased. Thus, when fertilizing with pig slurries, it is possible to match agronomic and mid-term soil chemical quality if the applied rate is adjusted to the potential productivity of the agricultural system.

## Data Availability

This paper contains the majority of the data collected or analyzed during the whole experimental period.

## References

[CR1] AENOR (2018) Alimentos para animales. Métodos de muestreo y análisis. Determinación de elementos traza, metales pesados y otros elementos en los alimentos para animales por ICP-MS (UNE-EN 17053). Asociación Española de Normalización, Madrid, Spain (In Spanish)

[CR2] Albuquerque DCK, Scheffer-Basso SM, Escosteguy PAV (2017). Residual effect of pig slurry on common carpet grass pasture. Rev Bras Eng Agric e Ambient.

[CR3] Allen RG, Pereira LS, Raes D et al (1998) Crop evapotranspiration - guidelines for computing crop water requirements - FAO Irrigation and drainage paper 56. Irrig Drain Pap No 56, FAO 300. 10.1016/j.eja.2010.12.001

[CR4] Anderson OE, Ohki K (1977). Manganese - the dependent element. Fert Solutions.

[CR5] Bai Z, Li H, Yang X, Zhou B (2013). The critical soil P levels for crop yield, soil fertility and environmental safety in different soil types. Plant Soil.

[CR6] Barber SA (1995). Soil nutrient bioavailability: a mechanistic approach.

[CR7] Bosch-Serra ÀD, Yagüe MR, Teira-Esmatges MR (2014). Ammonia emissions from different fertilizing strategies in Mediterranean rainfed winter cereals. Atmos Environ.

[CR8] Bosch-Serra ÀD, Yagüe MR, Poch RM (2014). Aggregate strength in calcareous soil fertilized with pig slurries. Eur J Soil Sci.

[CR9] Bosch-Serra ÀD, Ortiz C, Yagüe MR et al (2015) Strategies to optimize nitrogen efficiency when fertilizing with pigslurries in dryland agricultural systems. Europ. J. Agronomy. http://dx.doi.org/10.1016/j.eja.2015.03.003

[CR10] Bosch-Serra ÀD, Yagüe MR, Poch RM et al (2017) Aggregate strength in calcareous soil fertilized with pig slurries. Eur J Soil Sci. 10.1111/EJSS.12438

[CR11] Chaney RL (1989) Toxic element accumulation in soils and crops: protecting soil fertility and agricultural food-chains. In: Bar-Josef B, Barrow NJ, Goldshmid J (eds) Inorganic contamination of the vadose zone. Springer Ecol Stud 74: 140–158

[CR12] Cottenie A (1980) Soil and plant testing as a basis of fertilizer recommendation. FAO Soils Bulletin 38/1. Food and Agriculure Organization of The United Nations, Rome, Italy. https://www.fao.org/3/ar118e/ar118e.pdf. Accessed 4 January 2022

[CR13] da Veiga M, Pandolfo CM, Balbinot Junior AA, Spagnollo E (2012). Chemical attributes of a Hapludox soil after nine years of pig slurry application. Pesqui Agropecu Bras.

[CR14] de Oliveira DA, Pinheiro A, da Veiga M (2014). Effects of pig slurry application on soil physical and chemical properties and glyphosate mobility. Rev Bras Cienc Solo.

[CR15] Edmeades DC (2003). The long-term effects of manures and fertilisers on soil productivity and quality: a review. Nutr Cycl Agroecosystems.

[CR16] EU Nitrogent Expert Panel (2015) Nitrogen Use Efficiency (NUE) - an indicator for the utilization of nitrogen in agriculture and food systems. Wageningen University, Alterra, Wageningen, Netherlands. http://www.eunep.com/wp-content/uploads/2017/03/Report-NUE-Indicator-Nitrogen-Expert-Panel-18-12-2015.pdf. Accessed 8 April 2022

[CR17] Eurostat (2021) Pig population-annual data. https://appsso.eurostat.ec.europa.eu/nui/show.do?dataset=apro_mt_lspig&lang=en. Accessed 8 April 2022

[CR18] Generalitat de Catalunya (2019) Decreto 153/2019, de 3 de julio, de gestión de la fertilización del suelo y de las deyecciones ganaderas y de aprobación del programa de actuación en las zonas vulnerables en relación con la contaminación por nitratos procedentes de fuentes agrarias. DOGC 7911. https://vlex.es/vid/decreto-153-2019-3-797758021. Accessed 8 April 2022

[CR19] Greenwood DJ, Draycott AN (1989). Experimental validation of an N-response model for widely different crops. Fertil Res.

[CR20] Grohskopf MA, Correa JC, Cassol PC (2016). Copper and zinc forms in soil fertilized with pig slurry in the bean crop. Rev Bras Eng Agric e Ambient.

[CR21] Hendershot WH, Lalande H, Duquette M, Carter MR, Gregorich EG (2008). Ion exchange and exchangeable cations. Soil sampling and methods of analysis.

[CR22] Hughes S, Reynolds B, Bell SA, Gardner C (2000). Simple phosphorus saturation index to estimate risk of dissolved P in runoff from arable soils. Soil Use Manage.

[CR23] Jakubus M, Dach J, Starmans D (2013). Bioavailability of copper and zinc in pig and cattle slurries. Fresenius Environ Bull.

[CR24] Jones RJA, Hiederer R, Rusco E et al (2004) The map of organic carbon in topsoils in Europe. EUR 21209 EN. 2004. JRC28299. https://publications.jrc.ec.europa.eu/repository/handle/JRC28299. Accessed 18 December 2021

[CR25] Kowalski Z, Makara A, Fijorek K (2013). Changes in the properties of pig manure slurry. Acta Biochim Pol.

[CR26] Kumaragamage D, Akinremi OO, Racz GJ (2016). Comparison of nutrient and metal loadings with the application of swine manure slurries and their liquid separates to soils. J Environ Qual.

[CR27] Lindsay WC (1972) Zinc in soils and plant nutrition. Adv Agron. 10.1016/S0065-2113(08)60635-5

[CR28] Littell RC, Milliken GA, Stroup WW (1996). SAS system for mixed models.

[CR29] López-Alonso M, García-Vaquero M, Benedito JL (2012). Trace mineral status and toxic metal accumulation in extensive and intensive pigs in NW Spain. Livest Sci.

[CR30] Mahmood F, Khan I, Ashraf U (2017). Effects of organic and inorganic manures on maize and their residual impact on soil physico-chemical properties. J Soil Sci Plant Nutr.

[CR31] MAPA, Ministerio d Agricultura, Pesca y Alimentación (1990) Real Decreto 1310/1990, de 29 de octubre, por el que se regula la utilización de los lodos de depuración en el sector agrario. BOE-A-1990–26490. (in Spanish) https://www.boe.es/eli/es/rd/1990/10/29/1310. Accessed 8 April 2022

[CR32] Martínez E, Maresma A, Biau A (2017). Long-term effects of pig slurry combined with mineral nitrogen on maize in a Mediterranean irrigated environment. F Crop Res.

[CR33] McGill WB, Figueiredo CT, Carter M (1993). Chapter 22. Total nitrogen. Soil sampling and methods of analysis.

[CR34] Montaghian HR, Hosseinpur AR (2015). Rhizosphere effects on Cu availability and fractionation in sewage sludge-amended calcareous soils. J Plant Nutr Soil Sci.

[CR35] Montibeller F, Silva JA, Brasil M, do Amaral Sobrinho N, CalderínGarcía A (2017). Mitigation of heavy metal contamination in soil via successive pig slurry application. Soil Sediment Contam.

[CR36] Morera MT, Echeverría J, Garrido J (2002). Bioavailability of heavy metals in soils amended with sewage sludge. Can J Soil Sci.

[CR37] Mortvedt JJ (2005) Heavy metals in fertilisers: their effect on soil and plant health; Proceeding No.575.The International Fertiliser Society, Cambridge, UK

[CR38] Olsen SR, Watanabe FS (1963). Diffusion of phosphorus as related to soil texture and plant uptake. Soi Sci Soc Am Proc.

[CR39] Ovejero J, Ortiz C, Boixadera J (2016). Pig slurry fertilization in a double-annual cropping forage system under sub-humid Mediterranean conditions. Eur J Agron.

[CR40] Page AL, Chang AC, Sposito G, Pettygrove GS, Asano T (1981). Trace elements in wastewater: their effects on plant growth and composition and their behavior in soils. Irrigation with reclaimed municipal wastewater - a guidance manual.

[CR41] Pansu M, Gautheyrou J (2003a) Chapter 29. Phosphorus. In: Pansu M, Gautheyrou J (eds.) Handbook of Soil Analysis. Mineralogical, Organic and Inorganic methods. Springer Netherlands, Dordrecht, The Netherlands, p 809

[CR42] Pansu M, Gautheyrou J (2003b) Chapter 17. Carbonates. In: Pansu M, Gautheyrou J (eds.) Handbook of Soil Analysis. Mineralogical, Organic and Inorganic methods. Springer Netherlands, Dordrecht, The Netherlands, pp 141–142

[CR43] Porta Casanellas J, López Acevedo Requerin M, Rodríguez Ochoa R (1986) Técnicas y experimentos en Edafología. Colegio Ofcial de Ingenieros Agrónomos de Cataluña, Barcelona, Spain (in Spanish)

[CR44] Provolo G, Manuli G, Finzi A (2018). Effect of pig and cattle slurry application on heavy metal composition of maize grown on different soils. Sustain.

[CR45] Qaswar M, Yiren L, Jing H (2020). Soil nutrients and heavy metal availability under long-term combined application of swine manure and synthetic fertilizers in acidic paddy soil. J Soils Sediments.

[CR46] Reuter RJ, Heard TG, Alston AM (1973). Correction of manganese deficiency on barley crops on calcareous soils. I. Manganous sulphate applied at sowing and as foliar sprays. Aust J Exp Agric Anim Husb.

[CR47] Rodríguez Martín JA, López Arias M, Grau Corbí JM (2009) Metales pesados, material orgánica y otros parámetros de los suelos agrícolas y de pastos de España. Ministerio de Medio Ambiente y Medio Rural y Marino/Instituto Nacional de Investigación y Tecnología Agraria y Alimentaria, Madrid (in Spanish)

[CR48] SAS Institute (2014) Statistical Analysis System, SAS/TAT. Software V 9.4 SAS Institute, Cary, NC, USA

[CR49] Serrano-Barrientos EM (2001) Aplicación agronómica de purín de cerdo y de un polielectrolito: Efectos en el cultivo de ryegrass y en las aguas de drenaje. Dissertation, Curso Internacional de Edafología y Biología Vegetal, CSIC - Instituto de Recursos Naturales y Agrobiología de Sevilla (IRNAS), Sevilla, Spain (in Spanish). https://digital.csic.es/handle/10261/97909. Accessed 8 April 2022.

[CR50] Shakoor A, Shakoor S, Rehman A (2021). Effect of animal manure, crop type, climate zone, and soil attributes on greenhouse gas emissions from agricultural soils—a global meta-analysis. J Clean Prod.

[CR51] Soil Survey Staff (2014) Keys to soil taxonomy, 12th ed. USDA-Natural Resources Conservation Service, Washington, DC. https://www.nrcs.usda.gov/wps/portal/nrcs/detail/soils/survey/class/taxonomy/?cid=nrcs142p2_053580. Accessed 8 April 2022

[CR52] Suresh A, Choi HL, Oh DI, Moon OK (2009) Prediction of the nutrients value and biochemical characteristics of swine slurry by measurement of EC – electrical conductivity. Bioresour Technol. 10.1016/j.biortech.2009.05.006 10.1016/j.biortech.2009.05.006 19482470

[CR53] Tiecher TL, Ceretta CA, Comin JJ (2013). Forms and accumulation of copper and zinc in a sandy typic hapludalf soil after long-term application of pig slurry and deep litter. Rev Bras Cienc Solo.

[CR54] U.S. EPA (1996) Method 3052 (SW-846). Microwave assisted acid digestion of siliceous and organically based matrices. United States Environmental Protection Agency, Washington, DC, USA

[CR55] U.S. EPA (2007) Method 3051A (SW-846): microwave assisted acid digestion of sediments, sludges, and oils, revision 1. United States Environmental Protection Agency, Washington, DC, USA

[CR56] Valdez AS, Bosch-Serra ÀD, Yagüe MR (2020). Earthworm community and soil microstructure changes with long-term organic fertilization. Arch Agron Soil Sci.

[CR57] Walkley A, Black IA (1934). An examination of the Degtjareff method for determining soil organic matter, and a proposed modification of the chromic acid titration method. Soil Sci.

[CR58] Webster R, Lark (2018) Analysis of variance in soil research: let the analysis fit the design. Eur J Soil Sci. 10.1111/ejss.12511

[CR59] Webster R (2007) Analysis of variance, inference, multiple comparisons and sampling effects in soil research. Eur J Soil Sci.10.1111/j.1365-2389.2006.00801.x

[CR60] Zaragüeta A, Enrique A, Virto I (2021). Effect of the long-term application of sewage sludge to a calcareous soil on its total and bioavailable content in trace elements, and their transfer to the crop. Minerals.

